# Social environmental effects on gene regulation

**DOI:** 10.1007/s00018-013-1357-6

**Published:** 2013-05-18

**Authors:** Jenny Tung, Yoav Gilad

**Affiliations:** 1 Department of Evolutionary Anthropology, Duke University, Box 90383, Durham, NC 27708 USA; 2 Duke Population Research Institute, Duke University, Box 90420, Durham, NC 27708 USA; 3 Institute for Genome Sciences & Policy, Duke University, DUMC Box 3382, Durham, NC 27708 USA; 4 Department of Human Genetics, University of Chicago, 920 E. 58th Street, Chicago, IL 60637 USA

**Keywords:** Social stress, Social status, Early life adversity, Gene expression, DNA methylation

## Abstract

Social environmental conditions, particularly the experience of social adversity, have long been connected with health and mortality in humans and other social mammals. Efforts to identify the physiological basis for these effects have historically focused on their neurological, endocrinological, and immunological consequences. Recently, this search has been extended to understanding the role of gene regulation in sensing, mediating, and determining susceptibility to social environmental variation. Studies in laboratory rodents, captive primates, and human populations have revealed correlations between social conditions and the regulation of a large number of genes, some of which are likely causal. Gene expression responses to the social environment are, in turn, mediated by a set of underlying regulatory mechanisms, of which epigenetic marks are the best studied to date. Importantly, a number of genes involved in the response to the social environment are also associated with susceptibility to other external stressors, as well as certain diseases. Hence, gene regulatory studies are a promising avenue for understanding, and potentially developing strategies to address, the effects of social adversity on health.

## Introduction

The social environment has a clear and profound impact on the health and wellbeing of humans and other social mammals. Adverse social environments have been associated with poorer responses to infection [1–4], slower wound healing rates [5, 6], accelerated cellular immunosenescence [7, 8], and altered cortisol regulation [1, 9–12]. They are also linked to susceptibility to a broad spectrum of infectious and noninfectious diseases [1–4, 13], with a particularly strong tie to cardiovascular disease (CVD) [14–18], the leading global cause of mortality [19, 20]. Together, the cumulative epidemiological impact of the social environment is striking [14, 21–23]. High quality social relationships, for instance, confer a 50 % increase in likelihood of survivorship across adult ages, irrespective of causes of death (excluding suicide)—an effect comparable in magnitude to that of well-established risk factors like smoking and heavy alcohol use [21]. Understanding the nature of social environmental effects, and alleviating their negative consequences, is therefore a major priority in human health.

Identifying the mechanisms through which social environments “get under the skin” [24] to impact physiology and health is a key part of meeting this challenge [25–27]. In humans, some of these effects arise from physical stressors correlated with social adversity, such as reduced access to health care and differences in the practice of “health habits” (such as smoking and physical activity) [24–26]. However, strong evidence suggests that the relationship between social adversity and health extends beyond differences in health care and health habits. For example, employment grade (a measure of social status) was correlated with marked differences in CVD risk among British civil servants, despite universal access to health care. In addition, only a third of status-related differences in CVD susceptibility were explained by the combined effects of health-related risk factors, including smoking, cholesterol levels, blood glucose levels, and blood pressure [14, 28, 29] (see [23] for similar results with respect to social isolation).

Social environmental effects on health are also strongly paralleled in animal models, in which health habit- and health care-related explanations are implausible. Social status and competition, for example, are linked to wound healing rates [5], lymphocyte count [30], and cardiovascular response [12] in wild baboons that experience natural patterns of disease and mortality. In model systems that enable experimental control of the social environment, social conditions are also known to shape brain and behavioral development as well as influence disease progression [31, 32]. Importantly, the association between social adversity and mortality risk observed in humans has been recapitulated both in natural and captive animal populations [33, 34], pointing to the general importance of social conditions among social mammals.

Together, these findings indicate that social conditions themselves generate physiological consequences relevant to health. In many instances, these effects are believed to be mediated by chronic social stress resulting from prolonged exposure to social adversity [35]. Hence, the effort to understand social environmental effects on health has largely been reframed as an effort to understand the biological mechanisms involved in sensing and responding to stressful social environments. This perspective has guided biological research on social environmental effects towards a strong focus on the limbic system of the brain, which is linked to fear, anxiety, and emotion, and to the hypothalamic–pituitary–adrenal (HPA) axis, which is a primary mediator of the stress response (reviewed in [36–38]; regions of the brain with particular relevance to social behavior, such as the amygdala and medial prefrontal cortex, are reviewed in detail in [39, 40]). As a result, we now know a great deal about how social conditions influence neurological and endocrinological pathways in the body. Indeed, diagnostic changes in these systems are now used as evidence of a physiological response to social adversity.

In contrast, we know much less about the molecular intermediaries that mediate social environmental effects on neurological, endocrinological, or immunological traits, especially at the level of gene regulation. Changes in gene regulation are likely involved in both sensing and shaping the cellular response to social environmental conditions. For example, glucocorticoids (GCs: steroid hormones closely tied to the stress response [36, 38, 41]) act in part by activating the transcription factor NR3C1 (the glucocorticoid receptor), which leads to widespread downstream changes in cellular gene expression profiles. GC-related gene expression changes can in turn shape organism-level traits, such as stress reactivity and immune defense, suggesting that a gene regulatory perspective may yield important new insight into the physiological consequences of social stress. However, although social effects on gene regulation have been studied in depth in other behavioral model systems, research on their role in humans and other social mammals has until recently been lacking.

This gap in our knowledge is swiftly being filled. In the last 5 years, data supporting a close tie between social conditions and gene regulation have rapidly accumulated [42] across multiple tissues, species, and social contexts. Although at an early stage, this work has already begun highlighting the role of gene regulatory changes in shaping social environmental effects on other traits, including behavior, immune defense, and disease susceptibility. Here, we review the current state of knowledge regarding the connection between gene regulation and social environmental variation. We focus specifically on social mammals, in which social conditions either directly capture (in studies of humans) or parallel important dimensions of the human social environment (for more general reviews of social dynamics and gene regulation in other species, particularly social insects, see [43–46]). Based on the findings to date, we evaluate the potential for a gene regulatory perspective to contribute to understanding social environmental effects on health. Finally, we conclude by outlining important future directions for the field.

### Evidence for social environmental effects on gene expression

The most common relationship reported between the social environment and gene regulation involves variation in steady-state transcript (mRNA) expression levels, a general measure of gene regulation that integrates across multiple underlying regulatory mechanisms. In contrast to the breadth of research on social environmental effects more generally [18, 36], the majority of work at the level of gene expression is concentrated in a small set of mammalian species and social contexts: human populations, laboratory rodent models (rats and mice), and captive rhesus macaques. Together, these studies highlight the potential importance of gene expression data for understanding the physiological consequences of social environmental variation.

### Human societies

Research conducted directly on human subjects has played an essential role in demonstrating the relevance of gene expression differences to understanding social environmental effects on human health and disease. Social stress has been associated with differential gene expression for hundreds of genes in a variety of social contexts, including self-reported loneliness [47], socioeconomic status [48–50], receipt of social support [51], and long-term provision of social support to others [52]. Together, these studies provide evidence that the major dimensions of social adversity connected with health—social status and social isolation—are also reflected in patterns of gene expression (at least in peripheral blood mononuclear cells, PBMCs, where the majority of studies have been conducted). In particular, adverse social environments have been consistently linked to upregulation of genes involved in inflammation and adrenergic signaling. In addition, predicted binding sites for transcription factors involved in the stress response are often found upstream of social environment-associated genes. For example, binding sites for the glucocorticoid receptor, which mediates the cellular response to HPA axis signaling, are enriched near genes linked to differences in social status and social isolation. A similar pattern is observed for NFkB, a transcription factor that plays a crucial role in the inflammatory response [47, 49, 50].

Studies in humans are limited, however, in their ability to test for causal relationships between social conditions and gene expression, in large part because it is unethical and impractical to experimentally manipulate the long-term social conditions experienced by human subjects [53]. The social environment may therefore simultaneously affect, as well as be affected by, differences in gene expression levels. For example, changes in immune-related gene expression are known to alter feeding and social behavior, and changes in the expression of signaling molecules in the brain also change an individual’s willingness to socially interact [54–56]. Substantial genetic and demographic heterogeneity in human populations also make it difficult to rule out other confounding factors. For example, genetic variation segregating among human populations can have a substantial impact on human gene expression variation [57, 58], including in pathways relevant to stress response. Ancestry-associated allele frequency differences between Africans and Europeans, for instance, affect the gene expression response to in vitro glucocorticoid treatment in cultured lymphoblast cells [59]. The effects of social environmental conditions (e.g., socioeconomic status) that are correlated with genetic background can thus be difficult to tease apart from the effects of genetic background. Indeed, while taking into account an overall estimate of genetic background is a commonly used approach to addressing this problem, variation in genetic background across the genome makes it an imperfect solution [60].

Alternatives to the cross-sectional study design have been useful in overcoming some of these limitations. The problem of inferring causality has been in part addressed by studies in which reverse causation is unlikely. For example, PBMC gene expression at 110 transcripts differs between adults from low versus high early life socioeconomic status (SES) backgrounds, despite no current differences in the SES of the adult subjects [50]. This design helps in inferring causality by taking advantage of experimental “randomization” of early life background in adult subjects: gene expression patterns in adults are unlikely to causally predict the SES of their parents in early life (but see [53]). Longitudinal study designs have also been useful, especially in controlling for population heterogeneity [37, 61]. For instance, Murphy and colleagues followed 147 adolescent girls for 2½ years during a period in which the risk of peer-mediated social rejection was high. Whole blood samples were obtained every 6 months, allowing the authors to investigate the effect of social rejection on changes in gene expression across time. By comparing repeated samples taken from the same study subjects, they were able to show upregulation of the transcription factor *NFkB* and its inhibitor, *I*-*kB*, following, but not preceding, episodes of social rejection. The temporal pattern revealed in this study, with changes in gene expression observed only after a socially adverse experience, is consistent with a causal effect of social rejection on gene expression levels (although one cannot rule out correlations between social rejection and other factors) [61]. This study, however, reported gene expression data only for two genes, making it impossible to evaluate what proportion of the hundreds of genes associated with social environmental effects fall in the same category. Indeed, to our knowledge, no prospective genome-wide studies of longitudinal changes in gene expression levels have yet been conducted in the context of social environmental variation in any system.

Thus, while studies in human populations suggest that gene regulation is important to understanding social environmental effects, they also raise new questions, in particular because human studies provide incomplete answers about the degree to which gene expression changes are direct consequences, as opposed to correlates, of the social environment. They also have not addressed the degree to which social effects on gene expression are plastic in response to environmental change, or the role of gene expression differences in shaping downstream organism-level phenotypes. To tackle these questions, animal models for human social behavior—particularly rodents and rhesus macaques—have been essential.

### Rodent models

Laboratory mice and rats have long served as the experimental workhorses for understanding mammalian physiology and development, driven in part by the availability of powerful molecular methods for manipulating specific cells, genes, and pathways. Mice and rats are also social animals, and their dependency on social interactions has positioned them as important models for understanding social environmental effects. Rodent models have been of particular importance in extending the relationship between the social environment and gene expression from peripheral tissues, which are readily accessible in humans and other primates, to the brain, which is not. Indeed, much of what we know about social environmental effects on gene regulation in the brain is the product of studies in rodents (but see also [62, 63]).

Through detailed molecular dissections of gene regulation in the brain, studies in rodents have emphasized the importance of social conditions for influencing key neural signaling molecules, some of which are involved in responding to multiple social contexts. To do so, variation in the social environment—most often, via social stress—is experimentally induced using one of several well-established paradigms (Table 1). For example, chronic social stress can be triggered in adult males by subjecting them to repeated social defeat, which is ensured by introducing the study subject into the established territory of a more aggressive, often larger, male [64]. The gene expression levels of several neural signaling molecules have been demonstrated to change as a result. Males who experience repeated social defeat exhibit reduced hippocampal expression of several isoforms of brain-derived neurotrophic factor (*bdnf*), which is involved in neuronal maintenance and growth [65], and increased expression of an upstream regulator of *bdnf*, *ΔFosB,* in the medial prefrontal cortex [66]. Socially defeated males also show increased expression of the corticotropin-releasing factor (*Crf*) gene in the hypothalamus, a change involved in inducing the HPA response to stress [67]. Other forms of social adversity induce similar changes in core signaling molecules, including social isolation (*kisspeptin 1:* [68]), mother–infant separation (*Avp:* [69]), and poor maternal care (measured in adult offspring; *bdnf*, *NR3C1, ESR1*; [70–72]). Together, these studies provide direct evidence that social conditions can causally impact gene expression.

**Table 1:** Common animal models for social environmental variation

Model	Species	Timing	Description	Representative gene regulatory studies
Dominance rank	Rhesus macaques	Adulthood	Experimentally imposed position in a linear sex-specific dominance hierarchy, with harassment directed from higher to lower rank positions	[82]
Maternal aggression	Rhesus macaques	Early life	Rates of receipt of aggressive behavior directed from mothers to offspring early in life	[150]
Maternal care (licking and grooming)	Rats	Early life	Rates of licking and grooming behavior experienced by pups in a sensitive post-natal period	[70, 72, 101, 102]
Maternal resource limitation	Rats	Early life	Cross-fostering to mothers with abundant versus limited resources; resource limited mothers exhibit rougher handling/abusive behavior	[71]
Nesting condition	Rats/mice	Early life	Early life in communal nests with multiple mothers and pups versus nests containing only a single mother and her pups	[73, 74]
Periodic mother-infant separation	Rats/mice	Early life	Intermittent (e.g., daily) periods of mother–pup separation into different housing conditions during the post-natal period	[69]
Rearing condition	Rhesus macaques	Early life	Early life rearing with mothers, same-aged peers, or surrogate mothers (with limited exposure to peers)	[77, 110]
Social defeat (nest defense)	Rats/mice (females only)	Adulthood	Social submission of the study subject in the face of elevated aggression from a pregnant or lactating female	
Social defeat (resident/intruder)	Rats/mice (males only)	Adulthood	Social submission of the study subject (e.g., by repeated introduction into the territory of a more aggressive male)	[56, 65–67, 107, 113, 154]
Social integration	Primates, other social mammals	Adulthood/early life	Strength of social bonds with conspecific group members (often measured using grooming or proximity)	[126]
Social isolation (individual housing)	Rats/mice	Adulthood	Housing in isolation instead of with a group of conspecifics	[68]

Rodent models have also helped to establish a link between socially mediated change in gene expression and phenotypic variation at an organismal level. For example, *bdnf* gene expression appears to be important in sensing and responding to a broad set of social environmental conditions, including social enrichment (e.g., communal nesting, in which multiple mothers co-rear their offspring; [73, 74]) as well as social adversity (e.g., chronic social defeat and early adversity; [56, 65, 66, 71]). Interestingly, *bdnf* gene expression levels under chronic stress conditions are also associated with the severity of the impact of chronic stress on mouse behavior. Krishnan and colleagues showed that variation in stress-induced *bdnf* gene expression predicted the degree of behavioral resilience after chronic social defeat, measured by resumption of social interaction with other mice. In other words, although induction of *bdnf* gene expression was a general feature of the response to social defeat, mice that were more willing to re-engage in social interactions induced *bdnf* more strongly (specifically in the nucleus accumbens, NAc, a primary target of dopamine signaling). Remarkably, experimental interventions that altered BDNF protein levels in the NAc were sufficient to shift mice from a susceptible phenotype to a resilient phenotype, and vice versa [54]. Thus, this example illustrates both the power of experimental models for isolating causal links between social conditions, gene expression, and phenotype, and the complexity and bidirectionality of the gene expression–social environment relationship. In this case, social stress-mediated changes in gene expression also reciprocally influenced patterns of social interaction.

### Rhesus macaques

Although mice and rats are dependent on social interactions, the social environments experienced by rodent models differ substantially from those experienced in species more closely related to us [75]. Like humans, rhesus macaques experience prolonged periods of early life dependency and maintain individually differentiated, long-term relationships within large mixed-sex social groups. These properties, along with the prevalence of rhesus macaques in captivity and their relatively close evolutionary relationship to humans (rhesus macaque and human last shared a common ancestor ~25 million years ago; [76]), have long made rhesus macaques important models for human behavior and sociality. Recently, research on this system has been extended to include work on the gene regulatory response to social stress. In captive settings in particular, researchers have used rhesus macaques to bridge between the strength of experimental systems for inferring causality, and a desire for increased ethological relevance to humans.

Experimental strategies for studying the social environment in rhesus macaques depend on randomizing study subjects across different long-term social conditions in order to measure the physiological outcomes of different treatments. In one such approach, captive rhesus macaques reared with their mothers under normal social conditions were compared to individuals reared either with same-aged peers or by “surrogates” (terry cloth-covered hot water bottles), with short daily periods of peer exposure [77, 78]. Later in life, animals from all three treatments were grouped together in order to specifically assess the effects of differences in early life conditions within a common adult environment. Peer-reared and surrogate peer-reared study subjects exhibited higher rates of illness, weight gain, and stereotyped behavior [78]. These phenotypic effects may be mediated, at least in part, by changes in gene expression [77]. Comparison of even a small set of animals from each early life-rearing condition (4–5 animals per condition) revealed marked differences between mother-reared macaques and peer- and surrogate peer-reared macaques later in life [77]. Specifically, for several hundred PBMC-expressed transcripts, two-fold (or higher) differences in gene expression differentiated the mother-reared (control) animals from those that experienced substantial early life adversity. In contrast, peer-reared and surrogate peer-reared animals differed very little.

A second experiment examined the effects of social status-induced social stress—a model for chronic social stress in humans, as well as an analogue for socioeconomic status [18]—on gene expression levels. Social status in rhesus macaques is mediated by sex-specific dominance ranks, which in females are usually tied to matrilineal structure: females tend to adopt dominance ranks below those of their mothers. However, ranks can be experimentally manipulated by forming new social groups of unrelated individuals, in which earlier introduction predicts higher rank. Within these groups, harassment and aggressive behavior are directed asymmetrically from higher- to lower-ranking females, leading to stress-related changes in endocrine regulation, white blood cell profiles, and feeding behavior [79–81]. Within this paradigm, individual dominance rank has been associated with gene expression level variation for a large set of genes, and dominance rank stands out as one of the primary predictors of global variation in gene expression levels [82]. Indeed, this relationship was found to be strong enough that gene expression data alone were sufficient to correctly classify most individuals by social rank. Specifically, high-ranking, middle-ranking, and low-ranking members of ten social groups could be discriminated with ~80 % predictive accuracy, emphasizing the widespread effects of dominance rank on gene expression levels across the genome [82].

Remarkably, despite differences in the sources of social stress, the platforms used to measure gene expression levels, and downstream data analysis, social adversity-linked genes identified in the two studies significantly overlap (hypergeometric test: *p* = 0.001; Table 2). This overlap dovetails with findings in both studies supporting enrichment of genes involved in the immune response and inflammation [77, 82], and suggests that gene expression responses to social adversity may be characterized by a shared general pattern (a possibility that motivates independent analyses in other social contexts). Together, this work provides useful context for interpreting the results of studies in humans, in which social conditions cannot be experimentally controlled. Some of the patterns in social environment-linked gene expression data from humans are broadly recapitulated in the experimental studies of rhesus macaques. Thus, social environmental associations with gene expression in human societies may also reflect, at least in part, the direct consequences of social conditions for gene expression.

**Table 2:** Overlap between genome-wide gene expression studies of social stress in PBMCs

	Social status (RM)	Social isolation (H)	Early social adversity (RM)	Early SES (H)
Social status (RM)	–	4,126	4,657	4,497
Social isolation (H)	10 *p* = 0.18	–	10,792	10,581
Early social adversity (RM)	12 *p* = 1.16 × 10^−3^	5 *p* = 1.48 × 10^−5^	–	12,805
Early SES (H)	3 *p* = 0.83	5 *p* = 5.22 × 10^−6^	4 *p* = 1.42 × 10^−5^	–

Number of genes that were measured in both studies is provided above the diagonal; number of overlapping genes called significant in both studies is provided on the lower diagonal, along with the *p* value from a hypergeometric test assessing the probability of an overlap of the observed magnitude or greater. Low overlapping numbers can reflect significant overlap because a relatively small set of individual gene IDs were significantly associated with the social environment for several of these studies. Genes measured in each study were obtained from GEO accession numbers for the respective study (GSE34129 for rhesus macaque social status, GSE7148 for human social isolation, GSE35850 for rhesus macaque early social adversity, and GSE15180 for human early SES). Transcript IDs were translated to gene IDs based on Illumina [50, 82] or Affymetrix [47, 77] annotations for the array platforms used. Significance calls were based on the criteria used in each study

*RM* rhesus macaque, *H* human

### Scope of social effects on gene expression

The cumulative evidence for the association of the social environment with variation in gene expression indicates three general patterns. First, at least some correlations between social exposures and gene expression levels reflect causal effects. Second, associations of this nature appear to be important in a broad set of social mammals. Third, such associations commonly arise as a consequence of the major sources of social adversity previously described in human populations, such as social isolation and differences in social status.

However, the extent and generality of these effects remain poorly defined. Thus, we do not yet know whether social environmental conditions explain a large fraction of inter-individual variation in gene expression, similar to the effects of age, or whether they are instead specific to a smaller set of environmentally responsive genes. We also do not know what percentage of socially responsive genes we have captured thus far, or whether the genes we do know about respond specifically to socially induced stress, as opposed to stressful conditions in general.

In part, these gaps in our knowledge stem from the fact that, although many studies have focused on understanding sources of variation in mammalian gene expression (e.g., age, sex, genotype, and health status; [58, 83, 84]), they have not taken social environmental factors into account. Social variables are absent altogether for immortalized cell lines, where much work on human gene expression variation has taken place (especially with respect to genetic predictors of gene expression; [58, 85–87]). Additionally, social environmental conditions can be challenging to measure for living study subjects, and have historically been collected more often by social scientists than by researchers interested in functional genomics. Thus, if social environmental variation plays a role in gene expression measurements, its effects may often be treated in subsequent data analysis as experimental or technical noise.

At the same time, studies that focus explicitly on gene expression responses to social environmental factors remain limited to a few species, social contexts, and tissues. Even in animal models, we still know little about gene regulatory changes associated with the social environment under natural conditions (i.e., outside the laboratory or captive settings). We also know little about changes in tissues other than PBMCs and the brain, including organs involved in the stress response, such as the adrenal glands, or in diseases related to social adversity, such as the heart. Finally, the power to detect gene expression associations with social conditions has been limited in studies to date, which often focus on relatively small numbers of individuals. The responses documented thus far may therefore have captured only the genes that exhibit comparatively large effect sizes.

**Fig. 1:**
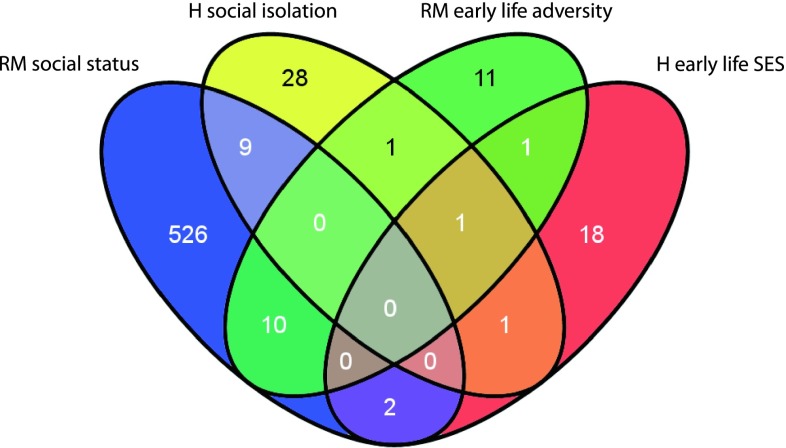
Overlap between significant social environment-responsive genes in PBMCs across four studies. The *Venn diagram* shows genes that were significantly associated with the social environment in each study, within the set of genes (*n* = 3,131) analyzed across all four studies. Note that the requirement that genes were included in all four studies substantially reduced the set of social environment-associated genes for each individual study (this was not a requirement for the pairwise analysis in Table 1)

Despite these limitations, it is possible to gain preliminary insight into the question of scope by aggregating evidence from comparable existing genome-scale studies. The best candidates for such an analysis are studies that focus on PBMCs, which have been profiled in both humans and rhesus macaques in response to several types of social adversity, including social status [82], social isolation [47], early life status [50], and early life-rearing condition [77]. Integrating the publicly available data from four such studies yields a set of 1,205 genes (5.4 % of the 22,132 unique genes included in these datasets) with evidence, in at least one study, of an association between gene expression and the social environment. Due to incomplete power and the technological limitations of the platforms used in these studies, this number is probably an underestimate. Thus, the available evidence suggests that social environmental effects can provoke widespread changes in gene expression levels, comparable to the effects of known physical stressors of human cells [88]. Additionally, a coherent, repeatedly detectable set of genes are involved in the response to social stress: genes associated with the social environment in one study tend to be enriched among the set of genes identified in other studies (Table 2), although the magnitudes of these overlaps is modest (probably due to the relatively small sets of genes identified in some studies, after conditioning on the set of genes tested across all studies; Fig. 1).

Consistent with the findings of each individual study, the aggregate set of social environment-associated genes is non-randomly distributed across functional categories. Immune system-related Gene Ontology (GO) categories are commonly overrepresented among these genes [89], including a number of gene sets involved in the response to pathogens. These changes may help account for differences in disease susceptibility related to the social environment, including evidence for differences in induction of pro-inflammatory genes following exposure to immune antigens [37, 50, 90]. The aggregate gene set also supports the importance of a few specific pathways. For example, genes involved in the response to glucocorticoids are enriched in this set, consistent with the inferred involvement of glucocorticoid regulation in several individual studies [47, 50, 82], and the role of GCs in the stress response more generally. Similarly, both the GO category “I-kappaB kinase/NF-kappaB cascade” and a set of genes regulated by NFkB transcription factor binding (based on TRANSFAC annotation) are also overrepresented [47, 50, 77]. These results raise the possibility that a small number of core pathways may account for the larger set of social environment-related changes in gene expression, an idea that would be consistent with results from rodents that emphasize the impact of one or a few key genes (e.g., *bdnf*).

Finally, this analysis highlights that it is possible to identify putatively functional gene expression responses to the social environment in peripheral blood cells. Specifically, social environment-dependent differences in immune function in these cells may help explain social mediation of health and disease susceptibility (for example, in response to infection by Epstein–Barr virus, respiratory infections, and the common cold; [2, 3, 91, 92]).

### Regulatory mechanisms that account for social environment-gene expression relationships

Changes in gene expression are themselves governed by changes in underlying gene regulatory mechanisms, which hold important clues to how social environmental effects arise and/or are maintained over time. Thus far, efforts to understand these mechanisms have focused primarily on epigenetic marks: DNA methylation and histone acetylation and methylation. This interest has been motivated by the apparent sensitivity of epigenetic marks to environmental conditions [93], including social adversity and chronic stress [94, 95]. In particular, a number of studies have focused on DNA methylation as a putatively stable gene regulatory mechanism with the potential to “encode” environmental experiences in the genome [96, 97].

A role for epigenetic marks in the gene regulatory response to the social environment has been embraced most strongly in research on the effects of social adversity in early life [98, 99]. Perhaps the most influential example of such an effect in mammals comes from work on maternal behavior and offspring stress reactivity in rats [72, 100]. Rat mothers engage in licking and grooming (LG) of their pups, but vary in the degree to which they perform LG behavior. In adulthood, the offspring of low LG mothers exhibit higher basal cortisol levels and greater cortisol secretion after acute stress, an effect that cross-fostering studies have linked to LG rates during the sensitive post-natal period. Stable changes in the regulation of the glucocorticoid receptor gene (*NR3C1*) in the hippocampus appear to account for these differences. Specifically, low rates of LG are associated with hypermethylation of the *NR3C1* promoter and reduced H3K9 histone acetylation. Together, these epigenetic changes interfere with binding of the transcription factor NGF1A, a driver of *NR3C1* gene expression [72].

This work has motivated a search for other epigenetically mediated effects of the social environment. Maternal LG exposure has now also been associated with long-term consequences for DNA methylation at the *Era1* estrogen receptor gene [101], as well as DNA methylation, histone acetylation, and histone methylation (H3K4me3) in the hippocampal glutamate receptor 1 (*Grm1*) gene [102]. The latter case suggests that stable epigenetic effects can be reversed in vitro: application of the demethylating agent 5-aza-cytidine successfully increased *Grm1* gene expression—usually depressed in low LG animals—in cultured hippocampal neurons [102]. Other early life environments are also associated with epigenetic changes. Periodic infant–mother separation, for example, results in hypomethylation of an enhancer associated with the vasopressin (*Avp*) gene in offspring. The resulting hypomethylated state persists into adulthood [69]. In humans, several studies have reported early life effects on the regulation of *NR3C1* that parallel those reported in rats. Among suicide victims for whom hippocampal slices could be obtained post-mortem, individuals who experienced child abuse also exhibited hypermethylated *NR3C1* promoter regions [62], although the pattern of differential DNA methylation initially identified appears to also extend more broadly in the genome [63].

Increasing evidence has linked epigenetic change to social environmental effects in adulthood as well as during early life, suggesting that epigenetic marks are often dynamically responsive to social environmental change. Evidence for epigenetic flexibility is available from all three mammalian systems in which social environmental effects on gene regulation have been studied (and is also consistent with evidence from other environmental exposures [103–106]). In mice, for example, both DNA methylation and H3K27 histone dimethylation patterns associated with *bdnf* gene expression are altered following chronic social defeat in adult males [65]. Additionally, social defeat precedes decreases in the expression levels of the histone deacetylase gene *HDAC5* [107], regulation of which has been shown to affect post-defeat patterns of social interaction [65]. In rhesus macaques, females assigned to high versus low social status in experimentally constructed groups are also distinguished by DNA methylation patterns [82], particularly in regions near genes that are differentially expressed in association with social status [82, 108]. Finally, in a recent population-based survey of 92 humans, current perceived stress levels as well as early life conditions (SES) were associated with DNA methylation at a similar number of sites genome-wide [90]. Thus, while epigenetic changes may be important in encoding early life social experiences, they also appear to be involved in the response to social conditions at other times in life. Indeed, studies of fear conditioning and physical activity have demonstrated that environmental effects often induce changes in the epigenetic landscape, including both passive and active (i.e., in the absence of cell division) changes [103–105]. Many of these responses, however, tend to be short-lived [104].

Together, these studies make a persuasive argument for the relevance of epigenetic mechanisms in shaping the gene regulatory response to variation in the social environment. However, few genome-wide analyses of epigenetic marks—and none, to our knowledge, on histone marks—have yet been published comparing individuals subjected to different social environmental conditions. Hence, we still know little about the general importance of epigenetic mechanisms in socially mediated gene regulation. With respect to DNA methylation, several array-based studies have scanned tens of thousands of CpG sites in the genome with mixed results. In humans, for example, although a larger number of CpG sites were associated with social stressors than expected by chance, only a few individual sites could be confidently associated with either early life SES or self-reported stress. The majority of these sites exhibited only modest effect sizes (<5 % difference in DNA methylation levels between high and low stress study subjects) [90]. Similarly, in two additional array-based studies (one on early life SES in humans and a second on early adversity in rhesus macaques), a large proportion of genes, including some that fall in biological pathways otherwise associated with social stress, exhibited evidence for differential DNA methylation for at least one probe. However, at the probe level, the number of probes associated with social environmental effects did not exceed the number expected by chance (i.e., the number expected from a uniform distribution of *p* values) [109, 110].

These findings suggest that the signal of social conditions in genome-wide studies of DNA methylation is subtler than for gene expression levels, consistent with the fact that DNA methylation is only one of a large number of mechanisms contributing to gene regulation. Interpretation of DNA methylation data is further challenged by the fact that we usually do not know how variation in DNA methylation levels translates to variation in gene expression levels, or which CpG sites are associated with regulation of a given gene. Genome-wide bisulfite sequencing to compare DNA methylation profiles in high and low status rhesus macaques illustrates this point. Although rank-related differentially methylated regions were enriched near differentially expressed genes, they often were too far from genes to be captured by most array-based approaches [82]. The functional significance of many social status-related differences in DNA methylation thus remains unclear.

### Gene regulatory plasticity in the face of social environmental change

Dissecting the mechanisms through which the social environment impacts physiology requires both identifying the biological pathways responsible for these effects and understanding the degree to which such effects are reversible. While some studies emphasize stable and enduring effects of social adversity—especially in relationship to poor social environmental quality early in life—other findings indicate substantial plasticity in social environment-mediated gene regulation. Indeed, at a basic level, associations between gene regulatory measurements and the social environment in adulthood alone suggest that individuals continue to flexibly respond to current conditions throughout life (e.g., [47, 51, 52]). However, correlations between early life social conditions and social conditions in adulthood—in other words, an individual’s long-term social history and accumulated social stress—are difficult to exclude for many cases.

The experimental evidence for plasticity is stronger. For example, in testing for the effects of experimentally imposed dominance rank in female rhesus macaques, samples were also opportunistically collected from a small subset of study subjects when they occupied different rank positions. For these females, gene expression profiles were therefore available from repeated samples collected before and after a rank transition. Interestingly, gene expression profiles from these samples strongly reflected the rank of the individual at the time of sampling, such that gene expression data correctly classified the majority of samples into the correct relative rank class (high, middle, or low) [82]. Thus, gene expression levels appear to rapidly track changes in social status. However, for rhesus macaques as well as humans, it remains unclear whether the apparent high degree of plasticity at the gene regulatory level also implies plasticity in the effects of gene regulation on downstream, organism-level traits.

To date, only studies in rodents clearly support this possibility. For example, although differences in DNA methylation in response to maternal LG behavior are laid down in early life and remain stable over time, they also appear to be reversible. Infusion of trichostatin A, a histone deacetylase inhibitor, both increased histone acetylation and decreased DNA methylation levels at *NR3C1*, resulting in erasure of stress reactivity differences between high and low LG offspring [72]. Similarly, depression of *bdnf* gene expression following chronic social defeat is mediated in part by local histone deacetylation. By targeted downregulation of a histone deacetylase gene (*HDAC5*), Tsankova et al. [65] showed that *bdnf* gene expression could be restored in socially defeated mice to levels characteristic of control, non-socially defeated mice. Further, intervention in *HDAC5* gene expression also affected the degree to which chronic social defeat reduced rates of social interaction. By chemically repressing *HDAC5* (using the antidepressant drug imipramine), social interaction rates could be restored to normal levels. Conversely, overexpression of *HDAC5* eliminated the effects of the drug [65].

Together, these studies demonstrate plasticity in the gene regulatory response to the social environment, at least in some cases. The extent of such plasticity remains to be determined, however, as strong evidence also supports canalization of gene regulation in other cases (e.g., [50, 52, 69, 71, 72, 101, 102]). Thus, neither universal stability nor unlimited plasticity is likely to characterize gene regulatory responses to the social environment. Rather than viewing these alternatives as conflicting, it will be valuable to investigate the conditions under which plasticity is favored or disfavored, including whether some responses are more plastic than others, and whether plasticity is associated with specific gene regulatory mechanisms. Similarly, variation in the severity, duration, or nature of social environmental exposures may be key. This possibility is supported by cases in which changes in gene regulation have been specifically linked to chronic social stress, but not acute social stress of the same type [65, 107].

### Relationship between social effects on gene regulation, health, and disease

One of the primary motivations for studying the biological mechanisms underlying social environmental effects stems from their dramatic impact on human health. While research on social conditions and gene regulation remains largely at a descriptive stage, it has progressed to a point where its utility in helping to address human health concerns can be evaluated. We see three indications that work thus far is relevant in this respect.

First, social conditions often affect the regulation of genes of known importance in disease risk or progression. This is apparent in analyzing the set of genes that respond to the social environment in PBMCs in conjunction with the NIA Human Common Disease gene sets, which are based on evidence of genetic association between genes and disease phenotypes in NIH’s Genetic Association Database [111, 112]. Fourteen disease-associated gene sets are overrepresented among social environment-correlated genes, at a false discovery rate of 5 % (Table 3) [89]. These gene sets include categories of genes that are broadly involved in disease progression and bacterial infection, as well as genes connected to individual conditions, such as *Helicobacter* infection and type II diabetes. Thus, social environmental conditions have the potential to perturb the expression of genes in which genetic perturbations are already linked to disease susceptibility.

**Table 3:** NIA human disease gene sets enriched among genes identified as responsive to social conditions in PBMCs

Gene set	FDR-adjusted *p* value	Expected number of genes	Observed number of genes
*Helicobacter* infection	0.002	2.56	10
Periodontitis	0.002	3.53	12
Bacterial infections and mycoses	0.006	8.10	18
Disease progression	0.006	12.18	24
Stomatognathic diseases	0.006	5.53	14
Acute disease	0.010	5.29	13
Disease susceptibility	0.011	5.53	13
Female urogenital diseases and pregnancy complications	0.011	10.42	20
Immune system diseases	0.011	6.25	14
Skin and connective tissue diseases	0.013	11.46	21
Squamous cell carcinoma	0.032	6.41	13
Stomach neoplasms	0.034	5.13	11
Diabetes mellitus, Type 2	0.050	16.59	25
Nutritional and metabolic diseases	0.050	28.86	39

Enrichments were calculated using the hypergeometric test implemented in [89] for categories with a minimum of 10 genes in the data set (the union set of genes identified as social environmentally responsive in [47, 50, 77, 82]). FDRs were calculated following the method of [155]

Second, in keeping with their role in health and disease susceptibility, social environmental exposures impact the response to external stressors, in part through affecting gene regulation. In mice, for instance, chronic social defeat blunts stimulation of the anti-apoptotic gene *bcl*-*2* during stroke, resulting in more extensive tissue damage in the brain [113]. In humans, PBMCs from individuals from low SES backgrounds induce higher levels of pro-inflammatory gene expression following stimulation with the immune antigens lipopolysaccharide, poly (I:C), and flagellin [37, 50] (see also [114] in rats). Additionally, glucocorticoid resistance—a physiological state associated with chronic stress as well as long-term treatment for some autoimmune disorders—can be predicted from gene expression profiles measured from isolated PBMCs, with over 80 % accuracy [115]. Thus, chronic stress appears to alter gene regulation in a manner that compromises reactions to subsequent stressors. Interestingly, even immortalization of primary cells appears to follow this pattern: cells from lonely individuals have been reported to require higher doses of Epstein–Barr virus for successful immortalization than cells from socially integrated individuals [92].

Finally, social conditions also predict gene expression patterns in tissues already affected by disease—thus providing a window into social environmental effects on disease progression as well as disease susceptibility. For example, social relationships have been suggested to predict cancer progression and outcome [13, 91]. Lutgendorf and colleagues showed that one potential avenue through which these differences arise is via gene expression patterns associated with variation in the tumor microenvironment (see also [116]). Specifically, they identified 266 transcripts that differentiated stage- and grade-matched ovarian carcinoma samples from women with high versus low social support. This set was enriched for genes involved in inflammation and beta-adrenergic signaling, reflecting increased tumor-specific noradrenaline levels in the low social support group. Similarly, T cell gene expression patterns (particularly in inflammation-related genes) differentiate asthmatic children from high SES versus low SES households, even after controlling for differences in use of asthma medication [49]. Because asthma medications often target pro-inflammatory pathways, the authors of this study argued that these differences suggest a potential effect of social adversity on the efficacy of asthma treatment. Hence, understanding the relationship between disease progression and gene regulatory responses to the social environment might also suggest therapeutic strategies for mitigating its negative effects.

### Future directions

Taken together, the cumulative evidence argues strongly in favor of a role for gene regulation in the response to social environmental conditions. In some cases, such as the response to early life social conditions in rodents, gene regulatory changes also clearly mediate the effects of social adversity on downstream behavioral, developmental, and health-related phenotypes. However, much remains unresolved about both the generality and context-dependence of these effects across different social contexts, as well as the gene regulatory mechanisms that that lie upstream of changes in gene expression levels. These complementary areas—one focused on breadth and context-dependence and the other focused on detailed molecular and mechanistic characterization—represent the next steps forward in linking social environmental variation to gene regulation.

In particular, while the existing data suggest that social conditions can exert widespread effects on gene expression, we know little about the factors that determine the severity and specific targets of social environmental effects [117], or about the extent to which these effects are plastic versus stable over time. We therefore cannot yet predict the social contexts or timing during which social environmental variation is likely to be important, how disparate social conditions affect different tissues, pathways, or regulatory mechanisms, or, most importantly, which individuals are more or less susceptible to adverse social conditions. For instance, although we know that age is a major predictor of gene expression variation, we do not know whether it alters the gene expression response to social stress. However, social adversity has been hypothesized to accelerate the aging process by targeting pro-inflammatory, oxidative stress, and telomere maintenance pathways [7, 8], suggesting that gene regulatory responses to social conditions may be highly age-dependent [118, 119]. Indeed, in non-mammalian systems such as honeybees, social cues can predict up to ten-fold differences in overall lifespan [120].

Genetic variation is also likely to affect the response to social stress. Gene–social environment interactions have been proposed by a number of candidate gene studies [121–126], including an influential (although controversial; [127]) set of studies arguing that susceptibility to depression and antisocial behavioral disorders results from the combination of susceptible genotype and adverse social conditions [123, 128]. At least in broad outline, these patterns (believed to be mediated by genetic effects on gene expression; [129, 130]) are consistent with experimental data on the stress response. For example, genotype is known to influence the gene expression response to stress-related GC treatment in cell lines [59] as well as *bdnf* secretion after social stress in male mice [54]. The generality and robustness of such interactions are an important direction for future work, as they are crucial for identifying the individuals who are most vulnerable to social adversity.

Conceptual frameworks for understanding variable susceptibility to social stressors are already available from the psychological literature, and may be helpful for motivating future sociogenomic studies. For example, some authors have argued that gene–environment interactions that are contingent upon timing of exposure (gene–environment–development interactions) will prove to be particularly important in shaping adult traits. Indeed, recent evidence demonstrates that a combination of prenatal and peripubertal stress, but not either alone, influences the amount of IL-1B and TNFa protein in the mouse hippocampus (genotype effects were not explored) [131]. Evolutionary psychologists have also argued that genetic variation in the response to social stressors may be maintained by selective mechanisms. For example, highly stress-reactive individuals (termed “orchids”) may endure the greatest fitness costs in adverse developmental environments but reap unusual benefits, relative to less stress-reactive “dandelions”, under favorable conditions [132, 133]. Genomic approaches aimed at identifying gene–environment interactions at the level of gene regulation, in association with sequence-based tests for a history of selection, could potentially help test this hypothesis. Challenges for such studies will include the high multiple testing burdens associated with genome-wide studies of interaction effects, as well as the need to control for genetic effects that also reciprocally influence social interactions [134, 135].

Understanding the conditions under which social stressors are most closely linked to gene regulation will also benefit from comparative studies in a more diverse set of species and social contexts [117]. An increasing number of studies tie social conditions to reproductive success, mortality rates, and other fitness-related traits, indicating that the scope for comparative approaches is broad [18, 33, 136–143]. Previous research on glucocorticoid levels and social status illustrates the utility of such approaches. Across social mammals, low social status is most closely associated with glucocorticoid levels in species in which social hierarchies are strictly enforced and sources of social support (particularly kin) are lacking [144]. Meanwhile, within species, social status effects appear to be most pronounced during periods of social instability [145, 146] (but see [11]). Social environmental effects on glucocorticoid regulation have also been reported to differ between captive and free-ranging animals—a contrast that may also affect baseline gene expression levels (e.g., [147]). In addition to investigating other social mammals, therefore, it will also be useful to expand current studies to include the effects of natural social environmental variation and unmanipulated behavioral patterns ([75]), including the consequences of social enrichment as well as social adversity [73, 74, 148, 149]. In a rare example of this approach, Kinnally et al. showed that variation in maternal aggression levels predicted expression of the serotonin transporter gene in infant rhesus macaque PBMCs (this was the only gene studied) [150]. Studies of this nature have the potential to complement work that relies on experimental manipulation, which often focuses on more extreme levels of social challenge.

With regards to molecular mechanism, studies in laboratory rodent models have clearly led the way. However, if we are concerned about the role that social environmental effects play in human health, better dissection of the regulatory mechanisms involved in the gene expression response to social adversity in humans (and other closely related animal models) is an imperative. The rapid development, increased sensitivity, and falling costs of new genomic approaches for measuring gene regulatory phenotypes should help in this regard, expanding the scope of social environmental effects on gene regulation to other gene regulatory mechanisms. For example, new approaches will facilitate testing hypotheses already developed in the published literature, such as whether GR or NFkB transcription factor binding events mediate many gene expression–social environment associations. Instead of inferring the presence of predicted binding sites based on gene expression data, these events can be directly measured on a genome-wide scale, including across different environmental contexts (e.g., glucocorticoid stimulation; [151–153]). In addition, experimental manipulation in cell culture provides a promising avenue for mechanistic dissection of potential gene regulatory mechanisms, given that work thus far supports retention of social environmental signatures in primary cells [92, 114, 115].

Finally, the limited ability to sample human and nonhuman primate tissues means that our understanding of socially mediated gene regulation in the periphery will likely increase more rapidly than in other important tissues, like the brain. While opportunistic sampling (as in [62]) should eventually help in this regard, it will be important to also develop a better understanding of the degree to which social environment-associated gene regulatory changes are general across tissues. A recent study using limited samples of T cells and prefrontal cortex from the same individuals, for example, suggests that social effects on DNA methylation may be broadly similar in both tissues [110]. However, extensive evidence of tissue-specific responses to social stress in mice suggests that this pattern will not always hold. More likely, responses to the social environment in different tissues will provide different, but complementary, insights into how social cues shape gene regulation. For example, while studies in the brain will be important for understanding the process of sensing and integrating the response to the social environment, studies in immune system-related cells, such as PBMCs, may be more useful for understanding its relationship to infectious disease.

## Conclusions

Strong evidence now links social environmental conditions with changes in gene regulation, a relationship that parallels and extends the effects of the social environment on other physiological traits. Measures of social status or social isolation are associated with changes in gene expression for a substantial fraction of the genome, although data from rodents suggests that the driving forces responsible for these changes may fall within a few core pathways. It is now incumbent upon researchers in this area to develop a more precise and nuanced understanding of the conditions under which social environmental effects on gene expression are most important. In doing so, we believe there is substantial potential to help address persistent questions about how social conditions influence disease risk and human health.
